# Interactions between alveolar epithelial cells and neutrophils under pro-inflammatory conditions

**DOI:** 10.3402/ecrj.v1.24545

**Published:** 2014-09-25

**Authors:** Ida von Schéele, Kjell Larsson, Lena Palmberg

**Affiliations:** Lung and Allergy Research, The Institute of Environmental Medicine, Karolinska Institutet, Stockholm, Sweden

**Keywords:** COPD, epithelial cells, neutrophils, Toll-like receptors

## Abstract

**Background:**

Intercellular communication is essential for defense and survival of the organism. The aim of the study was to find out whether there is an active crosstalk between airway cells constituting the first line of defense, alveolar epithelial cells (A549) and neutrophils, following activation with pro-inflammatory stimuli *in vitro* and to explore whether this communication is altered in chronic obstructive pulmonary disease (COPD), a condition characterized by chronic airway and lung inflammation.

**Methods:**

Blood neutrophils from healthy subjects and COPD patients were co-cultured with A549 cells in pure medium and in medium containing lipopolysaccharide (LPS), peptidoglycan (PGN), or tumor necrosis factor. The expression of Toll-like receptor 2 (TLR2), Toll-like receptor 4 (TLR4), and CD14 on the cell surface of neutrophils was assessed by flow cytometry, and release of CXCL8 (IL-8) and the soluble CD14 (sCD14) was measured in the supernatant with enzyme-linked immunosorbent assay (ELISA).

**Results:**

On neutrophils, the surface expression of TLR2 was diminished following activation with all three pro-inflammatory stimuli, and membrane bound (mCD14) and TLR4 expression were significantly increased in co-cultures compared to single cell cultures, irrespective of pro-inflammatory stimulation. There was a correlation between CXCL8 and sCD14 in LPS-stimulated co-cultured cells (*r*=0.82; *p*<0.01).

**Conclusion:**

An active crosstalk between A549 cells and blood neutrophils was clearly demonstrated, both in unstimulated cells and following activation with pro-inflammatory stimuli, *in vitro*. Co-culturing implied synergy and correlation between LPS-induced release of sCD14 and CXCL8, which indicates that sCD14 may be donated by neutrophils to epithelial cells facilitating TLR4-signaling. Furthermore, TLR2 on neutrophils was found to be down-regulated by pro-inflammatory stimuli.

The evolution of the human immune system has resulted in resistance to a variety of harmful organisms. The keystone of a functional host defense is the process of identification and elimination, which also includes communication between different specialized immune cell-types. A disrupted immune system may cause chronic inflammation and possible devastating effects in the host organism.

Chronic obstructive pulmonary disease (COPD) is associated with high morbidity and mortality (1) and is characterized by airway inflammation and tissue destruction with non-reversible airflow limitation. Today COPD is recognized as a systemic disease, thus not solely affecting the airways and lungs (2). Bacterial colonization of the lungs is often reported in patients with COPD, and is associated with more or less frequent acute exacerbations (3). Infiltration of neutrophils is characteristic of a variety of inflammatory disorders, and there is abundant evidence supporting that neutrophils are one of the main effector cells in COPD (4, 5). Neutrophils, like other phagocytes, have the ability to identify, phagocyte, and eliminate foreign particles and organisms by utilizing signals from, e.g. Toll-like receptors (TLRs). TLRs are highly conserved between species and currently, based on genomic analysis, 11 human TLRs have been identified (6). It is believed that each TLR recognizes a limited repertoire of pathogen-associated molecular patterns (PAMPs), but endogenous ligands are also found for some of the TLRs. Signaling through TLRs will lead to activation of the NF-кB pathway and thereby formation and release of pro-inflammatory cytokines and chemokines, e.g. interleukin (IL)-6, CXCL8 (IL-8), tumor necrosis factor (TNF), and Type 1 interferons (IFN) (7, 8). Toll-like receptor 4 (TLR4), one of the first TLRs to be discovered, has been extensively studied and responds, in a complex together with MD-2 (myeloid differentiation factor 2), to lipopolysaccharide (LPS) (9). This LPS–TLR4 interaction is enhanced by lipopolysaccharide-binding protein (LBP) and CD14, which have both been found in soluble form: sLBP and sCD14. Differences in epithelial cell CD14 expression (mCD14) seem to depend on the cell origin. Thus, alveolar epithelial cells are mCD14-negative and bronchial epithelial cells mCD14-positive (10). Toll-like receptor 2 (TLR2) recognizes lipoproteins from Gram-positive bacteria, and like signaling through TLR4, ligand binding to TLR2 leads to activation of the NF-кB pathway.

The epithelial layer functions as an effective barrier and has the ability to secrete a variety of effector molecules and thereby orchestrate the immune response of the host. The cooperation between neutrophils and epithelial cells is important as they both work within the first line of defense in the airways and on the alveolar levels. At co-culturing conditions of oral epithelium and neutrophils stimulated with a TLR2/4 agonist, TLR4 expression on epithelial cells increases, which seems to be dependent on polymorphonuclear leukocyte mediated regulation (11). Thus, neutrophils have the ability to protect the host indirectly through regulatory mechanisms of the innate immune receptors expressed by epithelial cells.

The aim of the study was to explore whether there is a bidirectional communication between alveolar epithelial cells and neutrophils during normal and pro-inflammatory conditions regarding TLR expression and regulation. Another aim was to find out whether a possible intercellular crosstalk may be impaired in neutrophils from patients with COPD compared to a control group of healthy non-smokers. Therefore, blood neutrophils from non-smoking healthy donors and smokers with COPD were co-cultured with alveolar epithelial cells (A549) in the absence and presence of pro-inflammatory stimuli.

## Materials and methods

Venous blood samples were drawn from 11 healthy, non-allergic, never-smokers [mean age 57 (range 49–64) years] and nine subjects with COPD [mean age 59 (range 48–67) years] defined as stage II or III according to GOLD criteria (mean FEV_1_ 68 (95% confidence interval 56–80) % of predicted value, mean FEV_1_/VC 0.57 (95% confidence interval 0.54–0.61)) (12) All participants in the COPD group were current smokers [mean 39 (range 15–60) pack-years]. All subjects gave their informed consent and the study was approved by the ethics committee at the Karolinska Institutet, Stockholm, Sweden.

### Cell-culture A549

The human alveolar epithelial cell line A549 (American Type Culture Collection, Rockville, MD; CCL185) were cultured in cell-culture flasks (Nunc, Denmark). The cells were provided with Ham's F-12 cell media (Gibco, Scotland, UK) supplemented with 1% penicillin/streptomycin (pest) (Gibco) and heat-inactivated fetal calf serum (FCS 10%) every second day (Gibco). At confluence the cells were detached by trypsin/EDTA and re-cultured in 24-well plates (Nunc). Passages 6–10 were used for the cell experiments.

### Isolation of neutrophils

We used a density gradient separation method for isolating blood neutrophils. Whole blood was mixed with an equal volume of Phosphate-buffered saline (PBS) (Gibco)-Dextrane 2% (Sigma-Aldrich, UK), and left for sedimentation. The leukocyte containing dextrane-blood was gently put on top of an equal volume of lymphoprep (Medinor AB, Sweden) and then centrifuged at 600 g for 25 min, without brake. The cell pellet was then resuspended in PBS, washed and then lysed in deionized water, and washed and finally resuspended in RPMI 1640 (Sigma-Aldrich) supplemented with 10% FCS, 1% L-glutamin, and 1% pest. Cell concentration and viability were established with Türc solution and Trypan blue (Sigma-Aldrich) and calculated in a Bürker-chamber. Viability as assessed by Trypan blue exclusion averaged >90% after 16 h of incubation. No significant difference in viability was noted between subject groups or treatment.

### Experimental design

A549 cells were cultured in a 24-well plate in supplemented F12 media. At semi-confluence (approximately 0.25 million cells/well), prior to co-culturing, the epithelial cells were stimulated with TNF (10 ng/ml, R&D), peptidoglycan (PGN 10 µg/ml, Sigma) or LPS from Escherichia coli 0111:B4 (LPS purified by gel-filtration chromatography, 1 µg/ml, Sigma) suspended in RPMI 1640 supplemented with 10% FCS, 1% L-glutamin, and 1% pest, 0.5 ml/well for 6 h.

Freshly prepared blood neutrophils were added to a cell-culture insert, pore size 0.4 µm (VWR) which does not allow the neutrophils to pass through the insert (13), and placed in each well of the 24-well plate (0.5 million/well), with or without stimulated or unstimulated A549 cells beneath the inserts. Adding neutrophils resulted in a dilution of the pro-inflammatory stimulus in each well, with a final concentration of TNF (5 ng/ml), PGN (5 µg/ml), and LPS (0.5 µg/ml) for the following 16 h of exposure. Each experiment was performed in duplicates with neutrophils from 11 healthy donors and from nine smokers with COPD. The supernatants were kept at −70°C until analysis. Neutrophils from each duplicate were pooled, washed, and analyzed for cell-surface expression of TLR2, TLR4, and mCD14 with flow cytometry.

### Flow cytometry

To analyze the expression of TLR2, TLR4, and mCD14, neutrophils were incubated with monoclonal antibodies for 30 min. (anti-TLR2-PE clone TL2.1, anti-TLR4-PE clone HTA125, anti-CD14-FITC clone 61D3, 10 µg/ml, eBioscience, San Diego, CA, USA). Isotype-matched antibodies were used as negative controls. After incubation the cells were washed twice and resuspended in PBS. Analyses were performed using FACSCalibur™ and median fluorescent intensity (MFI) was determined by CELLQuest™ (BD Bioscience Pharmingen) and calculated as relative median fluorescence intensity (rMFI=monoclonal antibody/corresponding isotype control).

### Soluble products

Soluble human CD14 (sCD14) was measured in the supernatants with a purchased DuoSet ELISA CD14 kit (R&D Systems, Europe, Abingdon, UK). The analysis was performed according to manufacturers’ protocol and the detection range was 62.5–4,000 pg/ml. For duplicate samples an intra-assay coefficient of variation (CV)<10% was accepted.

Interleukin-8 (CXCL8) was measured in the supernatants with in-house ELISA methods (14). Commercially available antibody pair MAB 208 and BAF 208 for detection of CXCL-8 (R&D systems, Europe, Abingdon, UK) was used as previously described. The detection range was 40–3,200 pg/ml. For duplicate samples, an intra-assay CV <10% was accepted.

### Statistics

Results are presented as mean±SEM. Comparisons between stimulations were performed by ANOVA (repeated measurement), followed by paired *t*-test when appropriate. Effects of co-culturing were tested with paired *t*-test between single- and co-cultured cells.

Between group comparisons were analyzed using ANOVA, followed by Fisher's Protected Least Significant Difference (PLSD) when appropriate, and Pearson's correlation coefficient. All data were analyzed by StatView version 5.0.1 (SAS Institute Inc., Cary, NC). A value of *p*<0.05 was considered significant.

## Results

### Surface expression of TLR2, TLR4, and mCD14

**Fig. 1:**
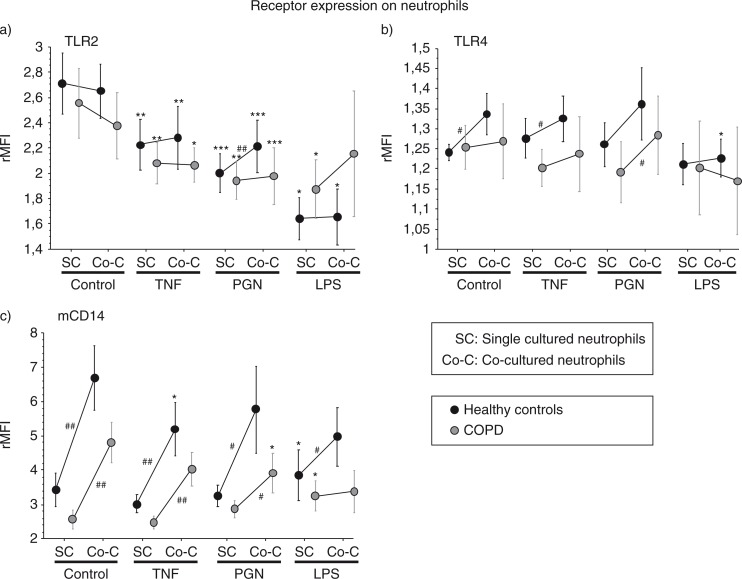
Cell-surface expression of TLR2 (a), TLR4 (b), mCD14 (c) on neutrophils, single cultured (SC) or co-cultured (Co-C) with A549 cells for 16 h, analyzed by flow cytometry. The neutrophils originated from healthy non-smokers (*n*=11) or COPD patients (*n*=9; for LPS stimulation *n*=5 for controls and *n*=6 for COPD). *p<0.05, **p<0.01, and ***p<0.001 compared with unstimulated cells. #*p*<0.05 and ##*p*<0.01 indicate differences between singled cultured and co-cultured neutrophils. Data are presented as mean±SEM.

Compared with unstimulated cells the surface expression of TLR2 was attenuated on blood neutrophils from both groups (controls and COPD) following stimulation with TNF, PGN, and LPS, irrespective of co-culturing with A549 cells (Fig. 1a).

Compared with single cultured neutrophils, TLR2 expression was up-regulated during PGN stimulation in co-cultured neutrophils from healthy controls (*p*<0.01; Fig. 1a). Expression of TLR4 was enhanced by co-culturing the neutrophils with epithelial cells both in the control situation and during TNF stimulation in healthy donors, but also in the PGN-stimulated cells from COPD patients (*p*<0.05; Fig. 1b).

Expression of mCD14 was up-regulated by LPS in the single cultured neutrophils and down-regulated by TNF and PGN in neutrophils co-cultured with A549 cells (Fig. 1c).

The expression of membrane bound CD14 (mCD14) was increased in co-cultured compared to single cultured neutrophils, in healthy controls and subjects with COPD (Fig. 1c).

### Soluble CD14 and CXCL8

**Fig. 2:**
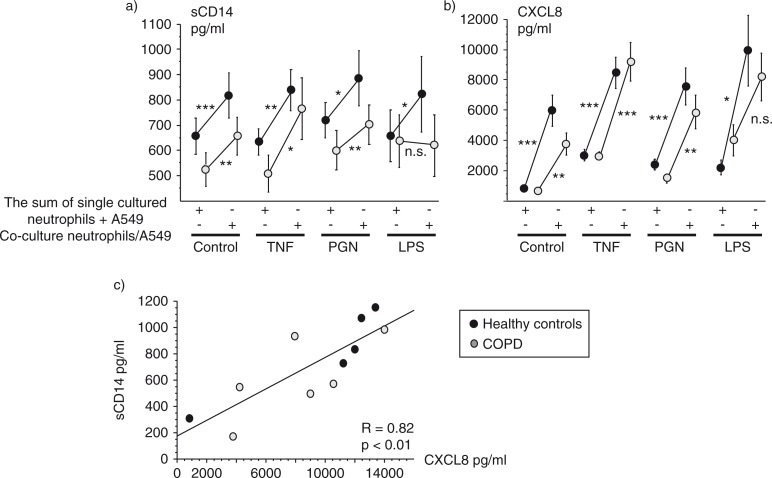
The sum of the concentrations of sCD14 (a) and CXCL8 (b) released by each cell type in single culture compared with the released amounts from co-cultured neutrophils and A549 cells measured with ELISA. Data are presented as mean±SEM. Regression plot of sCD14 and CXCL8 in LPS-stimulated co-cultured neutrophils and A549 cells (c). *p<0.05, **p<0.01, and ***p<0.001 indicate differences between single and co-cultured cells.

The release of sCD14 to the supernatant was unaffected by the three different pro-inflammatory stimuli (TNF, PGN, LPS) both in single cultured epithelial cells and neutrophils but also during co-cultured conditions (Fig. 2a, Table 1). There was one exception: LPS, which induced release of sCD14 from single cultured neutrophils (Table 1). All three pro-inflammatory stimuli elevated the release of CXCL8 into the supernatant, in A549 cells, neutrophils, and in the co-cultured situation. (Fig. 2b, Table 1). A positive correlation between sCD14 and CXCL8 release was found in unstimulated co-cultured cells (*r*=0.46; *p*=0.04, *n*=20). Stimulation with LPS induced a significant correlation between sCD14 and CXCL8 in co-cultured neutrophils and A549 cells (*r*=0.82; *p*=0.002, *n*=11; Fig. 2c).

**Table 1:** The effect of different stimuli (TNF, LPS, PGN) on A549 cells, neutrophils, and co-cultured cells on CXCL8 and sCD14 release, from healthy non-smokers and subjects with COPD

		CXCL8 (pg/ml)		sCD14 (pg/ml)	
			
		Healthy controls	COPD	Healthy controls	COPD
A549	Control	359±79	319±70	123±14	124±30
	TNF	2,141±313***	2,286±163***	114±19	127±28
	PGN	671±114***	556±84***	123±14	122±28
	LPS	350±81*	356±89*	85±18	114±40
Neutrophils	Control	476±133	359±103	534±71	401±44
	TNF	895±203*	722±136***	521±46	382±50
	PGN	1,735±343**	931±222**	598±74	478±57*
	LPS	1,849±457*	3,659±964*	573±91*	524±77**
Co-cultured	Control	5,964±1,111	3,765±701	818±90	657±76
	TNF	8,462±1,013*	9,188±1,273***	839±81	766±123
	PGN	7,568±1,199**	5,850±1,109**	885±111	703±78
	LPS	9,931±2,308	8,196±1,586*	823±150	620±123

The supernatants were analyzed with ELISA.

* *p*<0.05

** *p*<0.01

*** *p*<0.001 indicate differences between the pro-inflammatory stimulated cells and the medium controls.

To evaluate the effect of co-culturing, comparisons of the sum of the concentrations of sCD14 and CXCL8 released by each cell type in single culture was compared with the measured amounts released from co-cultured cells. Significantly higher levels of sCD14 and CXCL8 were found under co-culturing conditions, both in unstimulated and stimulated cells from healthy non-smoking controls and from the COPD group (Fig. 2a and b).

### Healthy controls vs. COPD patients

There were no significant differences between the healthy controls and the COPD patients with regard to any of the measured outcome variables.

## Discussion

This study demonstrated that the neutrophil surface expression of membrane bound CD14 and TLR4 increased when neutrophils were co-cultured with alveolar epithelial cells, irrespective of pro-inflammatory stimulation. Furthermore, it was shown that soluble CD14 (sCD14) and CXCL8 synergistically increased when neutrophils and alveolar epithelial cells were co-cultured, *in vitro* and that sCD14 strongly correlates to CXCL8 release during LPS stimulation of co-cultured cells, whereas no such effect was observed in the absence of epithelial cells. It is tempting to assume that these findings are a result of donation of sCD14 from neutrophils to A549 cells, facilitating TLR4-signaling in the otherwise CD14-negative A549 cells. Subsequently this will lead to elevated secretion of CXCL8 from A549 cells. These findings strongly support an active bidirectional crosstalk between neutrophils and epithelial cells.

Epithelial cells are known to produce very small amounts of both soluble and membrane bound CD14, a co-receptor that facilitates the signaling through TLR4. It has been reported that stimulation of A549 cells with LPS, PGN or *Klebsiella pneumonia* together with recombinant sCD14 increases the release of chemokines, such as CXCL8 compared with stimulation with LPS, PGN, and *Klebsiella pneumonia* alone (15–17). It thus seems likely that A549 cells, in our experiments, use sCD14, produced and released by neutrophils, as a co-receptor for TLR4 signaling. This will result in elevated CXCL8 production and release from the epithelial cells into the supernatant. Subsequently, this elevated CXCL8 production primes and activates neutrophils (18), which most likely yields in a self-amplifying mechanism with synergistically enhanced release of sCD14 and CXCL8. Thus, our results strongly indicate that there is an important interaction between neutrophils and epithelial cells that contributes to the maintenance of host defense.

The observed reduction of neutrophil TLR2-expression that was induced by the pro-inflammatory stimulation and not by co-culture conditions seems to be a general phenomenon, independent of which pro-inflammatory stimulus used, indicating both heterologous and autologous down-regulation. This observation is supported by studies on single cultured neutrophils and monocytes (19). It could be speculated that TLR2 expression on neutrophils, upon leaving the sterile environment in the circulation entering the site of inflammatory activity in the airways, is down-regulated as a counter-regulatory mechanism in order to avoid excessive cellular stimulation. This could occur either by internalization or release of the TLR2 receptor. Previous reports have demonstrated the presence of TLR2 in a soluble form, sTLR2 (20, 21). Moreover, a down-regulation of TLR2 has been shown on sputum neutrophils compared with blood neutrophils, as well as decreased expression of TLR2 in sputum neutrophils from COPD patients compared with a non-smoking control group (22). These data further confirm the *ex-vivo* findings of this study with a reduction of TLR2 expression under pro-inflammatory conditions.

TLR4 is only weakly expressed by neutrophils, compared to TLR2 and CD14, and it is known that stimulation with LPS increases mCD14 while leaving TLR4 expression unaffected in blood neutrophils (23). Therefore, it is intriguing that both mCD14 and TLR4 on neutrophils are up-regulated when co-cultured with A549, a mechanism that, unlike TLR2, seems to be independent of pro-inflammatory conditions. This indicates that an interaction between epithelial cells and neutrophils occurs, which involves regulatory mechanisms of the signaling pathway of TLR4. This novel finding needs further investigations to fully explain the underlying mechanisms by which epithelial cells contribute to the regulatory mechanisms of the host defense provided by neutrophils. It has been shown that infected epithelial cells have the ability to release heat shock protein (HSP), an endogenous TLR4 ligand, which activates neutrophils via TLR4. This activation of neutrophil results in increased release of TNF and CXCL8 (24). Probably endogenous ligands explain, at least partly, the observed synergistic effect on cytokine release and receptor regulation during co-culture conditions, observed in our study. But to what extent the results are dependent on a direct effect of exogenous ligands or a secondary and indirect effect of endogenous TLR-ligands is difficult to tell.

Stimulation with LPS up-regulates both mCD14 on neutrophils and sCD14 released by neutrophils. Previous studies have shown that sCD14 in bronchoalveolar lavage (BAL) fluid is elevated in smokers with and without COPD (25), who are continuously exposed to high concentrations of organic material including endotoxin (LPS) (26). Our findings strongly suggest neutrophils to be a potent donator of sCD14 to the surrounding environment, especially during LPS stimulation and in our case during co-culture conditions.

A limitation of the study is the somewhat small study sample, which influences the statistical power. Despite this, we found significant results both during pro-inflammatory and co-culturing conditions. However, there were no clear significant differences between COPD patients and non-smoking controls. We found, however, a consistent lower expression of TLR2 on neutrophils obtained from the COPD patients, irrespective of the stimulus, compared with healthy non-smokers. Thus, it seems most likely that the small, but entirely consistent, difference between healthy non-smokers and smokers with COPD indicates a real difference which did not reach statistical significance due to the small samples. In almost all measured outcomes, the COPD group shows an attenuated response compared with the control group. This may possibly reflect a reduction of innate immune responses, caused by chronic inflammation due to continuous exposure to pro-inflammatory stimuli.

In conclusion, we have shown that neutrophils release soluble mediators that act on alveolar epithelial cells (A549), but also that the epithelial cells influence the surface expression on several innate immune receptors on neutrophils. We therefore conclude that there is an active bidirectional crosstalk between epithelial cells and neutrophils during co-culture conditions. This interaction, which seems to facilitate identification and cellular response to LPS stimulation, is probably of importance in the immune response and contributes to the maintenance of the host defense.
